# Supplementary material for: Residential and wealth-related disparities of high fertility preferences in Ethiopia: A decomposition analysis
Source: PLoS One. 2024 Mar 7;19(3):e0299443. doi: 10.1371/journal.pone.0299443 (PMC10919854; doi:10.1371/journal.pone.0299443)
Supplement: Attachment — Submitted filename: Reviewer one comments.docx [file pone.0299443.s001.docx]

Residential and wealth-related disparities for high fertility preferences and it’s
contributing factors among reproductive-aged women in Ethiopia: a decomposition analysis.

General comments: The manuscript is generally interesting and with good insight. It will be better enough for comments and simply track sections if it could have page and line numbers.

Abstract:

**Method:**

1. I would stat the sample size in a second sentence.
2. I would put the outcome variable (the final sentences of the method) after the sample size or before the model as a third sentence.
3. What type of decomposition did you use for the second decomposition**?**

**Result**

1. I would put the CI for the fertility preference values.
2. You said that there is pro-poor distribution of fertility preferences, therefore it would be better if you list those categories of a variable that have a positive value for pro-poor distribution (pro-poor contribution) rather than simply listing significant variables.

**Methods**

1. It is good you wrote about Ethiopia’s healthcare delivery system, however, it would be better to focus on the level of the SRH service and preconception care.
2. **Please make sure about the following sentences. “**A total of 6134 women were included in the study population**” and “**After the exclusion,
   the final sample size used for the analysis was 13799**”.**
3. Would you specify the type of decomposition for the wealth-related inequalities?

**Result**

1. **Socio-demographic factors-** The first sentence (sample size) is already stated in the method.
2. **How can it possible to use
   marital status and husband occupation** /**education as variables in a single model. Because those who were not married have no husband.**
3. **Maternal health serve- “**The maternal health service-related variables applied to 6134 women for their recent children.**” Please double check this value.**
4. “Media exposure (17.5%), education (15.1%), marital status (8%), parity (7.4%), residence (3%) and region (2.2%) significantly contributed to the pro-poor disparity in high fertility preference among reproductive-aged group women in Ethiopia. Therefore, increasing media exposure and education among the poor population could lower the pro-poor distribution of high fertility by 17.5 and 15.1 percent (Table 5)”.

**This is your main outcome; therefore It would be better to state the contribution of the specific categories of the variable. Having media exposure increases education status, increasing parity, in rural residence, emerging region …** significantly contributed to the pro-poor disparity in high fertility

5. **Wealth was the significant contributor to residence-specific inequalities; however, residence is not a significant contributor to wealth-related inequalities. It is not a must but, would u double-check it.**

**Discussion:**

1. “Residential and socioeconomic disparity”. - It would be better to be Residential and wealth-related inequality in all sections of the manuscript. The other sociodemographic/socioeconomic factors are contributors, we are not sure about the inequalities of those variables.
