# Supplementary material for: Residential and wealth-related disparities of high fertility preferences in Ethiopia: A decomposition analysis
Source: PLoS One. 2024 Mar 7;19(3):e0299443. doi: 10.1371/journal.pone.0299443 (PMC10919854; doi:10.1371/journal.pone.0299443)
Supplement: Attachment — Submitted filename: Point by point response.docx [file pone.0299443.s002.docx]

Date: 15/01/2024

PLOS ONE

Editorial office

Subject: Manuscript revision for publication considerations

Dear Editor,

We thank the editor and reviewers for providing feedback on our manuscript. We have thoroughly addressed and incorporated all the comments and suggestions into the revised manuscript. We have also provided a point-by-point response to the reviewer's comments and indicated all changes in track changes.

We hope that you will find the revised manuscript acceptable for publication. Please do not hesitate to contact me for further information or clarification.

Thank you for considering our manuscript.

Best regards,

Melaku Birhanu

Corresponding Author

**A point-by-point response for the editor**

**Conclusion and recommendations:**

The provided recommendations are broad and might pose challenges for relevant authorities to implement based on the study's findings. Therefore, it would be beneficial if the authors could provide specific recommendations directed towards the relevant stakeholders.

***Response: Thank you for your suggestions. We have provided specific recommendations. Please take a look at Page 25 lines 141-147.***

The implication of the findings:

Summarize the practical significance of your findings. For instance, you can summarise how the observed findings impact existing practices or policies. This would consider the relevance and potential consequences of this study's findings.

***Response: We have added the practical significance of the study. Please look at Page 5, lines 10-16.***

Further, you could discuss the generalizability of your findings to other populations or settings. This could clarify the extent to which your results can be applied beyond the specific context of your study.

***Response: The study is*** ***applicable to low and middle-income countries with high fertility rates. We have mentioned it in the conclusion section.***

**A point-by-point response for the reviewer #1**

The manuscript is generally interesting and with good insight. The methdology section specfically data analysis technique was interesting and able to answer the research quastions It will be better enough for comments and simply track sections if it could have page and line numbers.

***Response: Thank you for your comments. We are sorry for the inconvenience. We have added a page and line number.***

**Abstract**

**Method:**

1. I would stat the sample size in a second sentence.

***Response: We have taken your comments and included the sample size (page 2 line 10-12). In addition, we have moved the sample size to the second paragraph (Page 6, line 24).***

2. I would put the outcome variable (the final sentences of the method) after the sample size or before the model as a third sentence.

***Response: We put the outcome variable next to sample size (page 2 line 10-12).***

3. What type of decomposition did you use for the second decomposition?

***Response: We used Erreygers' normalized concentration index to decompose the wealth-related disparity.***

**Result**

1. I would put the CI for the fertility preference values.

***Response: Thank you. we included the CI value ECI=-0.14, SE=0.012, on the abstract (page 2 line 50).***

2. You said that there is pro-poor distribution of fertility preferences, therefore it would be better if you list those categories of a variable that have a positive value for pro-poor distribution (pro-poor contribution) rather than simply listing significant variables.

***Response: We have corrected it by listing all significant variables that contribute to the pro-rich distribution (page 2, line 30)***

**Methods**

1. It is good you wrote about Ethiopia’s healthcare delivery system, however, it would be better to focus on the level of the SRH service and preconception care.

***Response: We have added the reproductive health and preconception care (page 6 line 8-10 and line 12-15)***

2. Please make sure about the following sentences. “A total of 6134 women were included in the study population” and “After the exclusion, the final sample size used for the analysis was 13799”.

***Response: Sorry for the confusion. The 6134 women are only for maternal health service (women with at least one child), but the 13799 are the total women included in the analysis.***

3. Would you specify the type of decomposition for the wealth-related inequalities?

***Response: We used Erreyger's normalized concentration index to decompose the wealth-related disparity.***

**Result**

1. Socio-demographic factors- The first sentence (sample size) is already stated in the method.

***Response: We have corrected the statement by removing the weighted sample size from the method section (page 7 line 20).***

2. How can it possible to use marital status and husband occupation /education as variables in a single model. Because those who were not married have no husband.

***Response: Thanks for your comment. We have removed the husband characteristics from the table (page 11).***

3. Maternal health serve- “The maternal health service-related variables applied to 6134 women for their recent children.” Please double check this value.

***Response: The 6134 women is only for maternal health service (women who have aleast one child)***

4. “Media exposure (17.5%), education (15.1%), marital status (8%), parity (7.4%), residence (3%) and region (2.2%) significantly contributed to the pro-poor disparity in high fertility preference among reproductive-aged group women in Ethiopia. Therefore, increasing media exposure and education among the poor population could lower the pro-poor distribution of high fertility by 17.5 and 15.1 percent (Table 5)”. This is your main outcome; therefore It would be better to state the contribution of the specific categories of the variable. Having media exposure increases education status, increasing parity, in rural residence, emerging region … significantly contributed to the pro-poor disparity in high fertility

***Response: Thank you for your recommendations. We have stated the contribution of primary education and corrected the in-text citations. Please look at page 19.***

5. Wealth was the significant contributor to residence-specific inequalities; however, residence is not a significant contributor to wealth-related inequalities. It is not a must but, would u double-check it.

***Response:*** ***We apologize for the typo mistake. The residence holds a significant value.***

**Discussion:**

1. “Residential and socioeconomic disparity”. - It would be better to be Residential and wealth-related inequality in all sections of the manuscript. The other sociodemographic/socioeconomic factors are contributors, we are not sure about the inequalities of those variables.

***Response: Thank you for your comment. Inequality is usually used for positive (e.g., health services uptake) that we would like to promote. Thus, we believe disparity could be a good fit for the area. Therefore, we have corrected it to “residential and wealth-related desparity”.***

**A point-by-point response for the reviewer #2**

Overall, it would be great if the authors put the page numbers and line numbers to easily cite the areas of concern.

***Response: Thank you for your comments. We are sorry for the inconvenience. We have added a page and line number.***

**A. Abstract**

1. The recommendations are general and could be difficult to take action by concerned bodies based on the findings of this study. Hence, it would be good if the authors put specific recommendations to relevant stakeholders (policymakers, planners, programmers and practitioners) to alleviate such issue.

***Response: Thank you for your suggestions. We have provided specific recommendations. Please take a look at Page 25 line 141-147.***

**B. Main document**

**Introduction**

1. The authors put a good background to the current study. It would be also great to put the existing initiatives by the government to address the problem in a paragraph (including policy, strategy, plans and service access, etc). For example, National population policy and reproductive health strategy of the country could be consulted.

***Response: We have added the policy and stratagies. We have also added the practical significance of the study. Please look at Page 5 lines 10-16.***

**Measurements of variables in the methods section**

1. The categorization of the region variable needs reference citation. It could have effect on both residence and wealth status indicators.

***Response: We have cited the reference for the region and media exposure. Please look at page 7, lines 7-9.***

2. The wealth index concept and its construction need more elaboration (particularly what it intends to measure and what type of data was used in its calculation). You could visit the DHS Program regarding wealth index construction and also cite the country’s 2016 DHS survey methodology.

***Response: We have added more about the wealth index. Please look at page 7, lines 13-15.***

**Data analysis**

1. Why the authors preferred to use the Erreygers normalized concentration index (ECI) in the presence of other similar indices such as the Wagstaff’s concentration index?

***Response: Wagstaff’s concentration index doesn't estimate the absolute inequality of health when the outcome is binary, and the ECI is the normalized version of Wagstaff’s concentration index, which corrects this limitation (explained in the method section).***

2. What STATA command did the authors use to calculate the concentration index? It would be worthwhile mentioning it to the reader.

***Response: We have added the STATA command. Please look at page 9 lines 18-19***

**Results**

**Decomposition of fertility preference across the residence**

1. “More than half (40.9%) of variations in high fertility preference between rural and urban residents were explained by the difference in composition (endowments) and the remaining differences (59.1%) were attributed to the difference in the effect of each variable for urban and rural residences (coefficients)”. The term “more than half” should be revised as 40.9% is not comparable with that.

***Response: We have corrected it. Please look at page 14 lines 2-5***

**Concentration curve and index**

1. It’s good reporting the values of concentration index with its standard error. It would be also great to incorporate the 95% confidence interval of the concentration index.

***Response: We have put the 95% confidence interval***

**Conclusions**

1. See the comment above in the abstract section related to the recommendations.

***Response: We have modified it accordingly.***
